# Effect of Ultrasound-Guided Subacromial Bursa Injections With Various Doses of Corticosteroid in Subacromial Bursitis: A Retrospective Study

**DOI:** 10.7759/cureus.89307

**Published:** 2025-08-03

**Authors:** R. Yatish, Ashok Kumar B. K., Abhinav A Suvarna, T. S. Channappa, Manju Jayaram, H. B. Shivakumar

**Affiliations:** 1 Orthopedics, Kempegowda Institute of Medical Sciences and Research Center, Bangalore, IND; 2 Pain Management, Kempegowda Institute of Medical Sciences and Research Center, Bangalore, IND

**Keywords:** corticosteroid injection, shoulder pain, subacromial bursitis, triamcinolone, ultrasound-guided injection

## Abstract

Background: Subacromial bursitis is a common cause of shoulder pain and functional limitation, due to inflammation of the subacromial bursa. Corticosteroid injections are widely used in cases unresponsive to conservative treatments. However, the optimal dose for achieving sustained symptom relief with minimal adverse effects, especially when administered under ultrasound guidance, remains a topic of clinical interest. This study evaluates the efficacy of different corticosteroid doses delivered via ultrasound-guided subacromial bursa injection.

Methodology: This retrospective study was conducted at a tertiary care center between May 2018 and June 2023. Patients aged 30-60 years with MRI-confirmed subacromial bursitis who failed conservative treatment for at least three months were included. Subjects were divided into two groups: a low-dose group receiving 10 mg triamcinolone with 1% lignocaine and a high-dose group receiving 20 mg triamcinolone with 1% lignocaine. Injections were administered under ultrasound guidance using a linear probe. Pain was assessed using the Numerical Rating Scale (NRS) at baseline, and at one week, one month, three months, and six months post-intervention. Data were analyzed using t-tests and chi-square tests.

Results: Both groups showed significant reductions in pain scores at all post-injection time points (*P* < 0.05). The high-dose group demonstrated more substantial and sustained improvement in pain relief, with mean NRS scores decreasing from 8.1 at baseline to 2.4 at six months, compared to a decline from 7.9 to 3.5 in the low-dose group. Adverse events were minimal and similar across both groups.

Conclusions: Ultrasound-guided corticosteroid injections represent a safe and effective treatment for subacromial bursitis, with higher doses of triamcinolone offering longer-lasting pain relief. The technique improves precision and minimizes risk, making it a valuable tool in managing refractory shoulder pain. Further prospective studies are recommended to validate these findings and develop standardized dosing protocols.

## Introduction

A large proportion of adults experience shoulder pain at some point in their lives, and subacromial impingement, which includes bursitis, is a common underlying pathology [1]. The subacromial bursa, a fluid-filled sac located between the acromion and the rotator cuff tendons, plays a crucial role in reducing friction during shoulder movement [2]. When inflamed, this bursa can cause significant pain, stiffness, and a restricted range of motion, which can negatively impact daily activities and quality of life [3].In addition, the burden of shoulder disorders, including subacromial bursitis, is growing in developing countries such as India, where aging populations and occupation-related shoulder strain are increasingly common. 

The shoulder joint is one of the most mobile and complex joints in the human body. The subacromial space, located between the acromion of the scapula and the head of the humerus, houses several important structures, including the subacromial bursa, supraspinatus tendon, and portions of the long head of the biceps tendon and joint capsule [4]. The subacromial bursa serves as a cushion to reduce friction between the rotator cuff tendons and the overlying acromion and deltoid muscle. Inflammation of this bursa, caused by repetitive motion, trauma, or impingement, results in bursitis, contributing to pain and limited mobility [5]. Understanding this anatomy is crucial when administering corticosteroid injections, as blind techniques may result in inaccurate drug placement, reduced efficacy, or injury to surrounding tissues. Ultrasound guidance facilitates real-time visualization of the bursa, improving the precision and safety of the intervention [6].

The etiology of subacromial bursitis is multifactorial, often linked to repetitive overhead movements, mechanical impingement, and degenerative changes within the shoulder joint [7]. Common risk factors include occupations that involve repetitive lifting, sports-related activities, and age-related degeneration [8]. Patients with subacromial bursitis often present with progressive shoulder pain, tenderness over the anterolateral aspect of the shoulder, and discomfort exacerbated by abduction and external rotation [9].

Management of subacromial bursitis typically involves conservative treatment modalities such as physical therapy, nonsteroidal anti-inflammatory drugs (NSAIDs), and activity modification [10]. However, in cases where these approaches fail to provide sufficient relief, corticosteroid injections are widely employed for their potent anti-inflammatory effects [11]. The use of ultrasound guidance has become increasingly popular in recent years, as it enhances the accuracy of drug delivery into the subacromial space and minimizes complications [12].

Several studies have examined the efficacy of corticosteroid injections for subacromial bursitis with varying outcomes based on the dose administered, the number of injections, and whether imaging guidance was used [13]. Although lower doses may offer temporary symptom relief, higher doses of corticosteroids are often associated with more prolonged benefits [14]. However, the optimal dose for sustained improvement without increased adverse effects remains unclear. Repeated or high-dose (HD) corticosteroid use may carry risks such as tendon weakening, subcutaneous fat atrophy, and systemic side effects, especially if not administered accurately [15].

There is a lack of region-specific evidence guiding optimal corticosteroid dosing strategies, particularly when using ultrasound-guided techniques. Given the widespread use of corticosteroid injections in orthopedic and pain clinics, it is essential to establish evidence-based dosing protocols that balance efficacy, safety, and cost-effectiveness [16].

Therefore, this study was conducted to evaluate the therapeutic effect of ultrasound-guided subacromial bursa injections with varying doses of corticosteroids in patients diagnosed with subacromial bursitis. By comparing pain outcomes over a six-month follow-up period, the study aims to provide clinically relevant data to inform practice and contribute to optimizing treatment strategies for this common musculoskeletal condition.

## Materials and methods

Study design and setting

This retrospective comparative study was conducted at the Department of Orthopedics and Pain Management, Kempegowda Institute of Medical Sciences and Research Center (KIMS and RC), Bangalore, between May 2018 and June 2023. The aim was to evaluate the therapeutic efficacy of two different doses of ultrasound-guided corticosteroid injections administered into the subacromial bursa.

Patient selection

Patients aged 30 to 60 years with a diagnosis of subacromial bursitis confirmed through MRI and X-ray imaging were screened for inclusion. All patients had experienced persistent shoulder pain and functional limitation for at least three months despite conservative treatment, including physiotherapy and NSAIDs. Numerical Rating Scale (NRS) [17] was used to measure pain levels pre- and post-intervention at specified intervals: one week, one month, three months, and six months.

Patient records were screened based on outpatient visit logs (from Medical Records Department), procedure notes, ultrasound reports, and MRI findings confirming subacromial bursitis. A purposive sampling method was employed, selecting only those who had complete documentation, met all inclusion/exclusion criteria, and had post-intervention follow-up data up to six months.

No formal power analysis was conducted due to the retrospective nature of the study. However, the sample size was deemed sufficient based on feasibility, completeness of follow-up data, and alignment with prior published studies evaluating corticosteroid injection outcomes in similar clinical settings.

Inclusion criteria

Patients aged between 30 and 60 years, with clinically diagnosed shoulder impingement syndrome confirmed via physical examination [18], radiological diagnosis of subacromial bursitis on MRI and X-ray imaging, failure of conservative treatment for at least three months, body mass index (BMI) < 30, non-diabetic status, and a normal coagulation profile were included in the study.

Exclusion criteria

Patients with rotator cuff tears or calcific tendonitis on imaging, adhesive capsulitis or post-traumatic shoulder pathology, active infection or skin lesions at the injection site, allergy to corticosteroids or local anesthetics, those on anticoagulant or antiplatelet therapy, and individuals with known bleeding disorders were excluded from the study.

Consent and ethical considerations

Written informed consent was obtained from all eligible patients before the intervention. The study was conducted by institutional ethical standards (Ref. no. KIMS/IEC/A318/M/2025).

This was a non-randomized, retrospective study, and no blinding was performed during the data collection phase. However, to enhance data reliability, pain scores and procedural details were extracted independently by two investigators from the medical records department. Any discrepancies were resolved by consensus or referred to a third senior reviewer.

Intervention procedure

Eligible patients were divided into two groups based on the dose of corticosteroid administered: The decision to administer either 10 or 20 mg of triamcinolone acetate was based on the treating clinician’s judgment, which considered factors such as symptom severity, chronicity, prior response to treatment, and patient tolerance. All procedures were performed by a single experienced interventional specialist using the ultrasound-guided technique and equipment.

Group high dose (HD): Triamcinolone acetate 20 mg + 1% lignocaine (total volume 3 mL)

Group low dose (LD): Triamcinolone acetate 10 mg + 1% lignocaine (total volume 3 mL)

All injections were performed under sterile conditions using real-time ultrasound guidance with a GE LOGIQ E machine (GE Healthcare, Chicago, IL) and an 11 MHz linear probe. Patients were positioned supine, and a 23-G spinal needle was used to access the subacromial bursa, confirmed via sonographic visualization.

Follow-up and outcome measures

All included patients had complete medical records with documented baseline characteristics, procedural details, and follow-up NRS pain scores at all predefined intervals: one week, one month, three months, and six months. Patients with incomplete follow-up data, missing pain scores, or insufficient procedural documentation were excluded during the initial screening. As a result, no imputation techniques were needed, and the final dataset comprised only complete cases (*n* = 64).

Pain levels were assessed using the NRS [17] at the following intervals:

Pre-intervention (baseline)

One week post-injection

One month post-injection

Three months post-injection

Six months post-injection

Statistical analysis was performed using IBM SPSS Statistics, version 26.0 (IBM Corp., Armonk, NY). Continuous variables, such as age, BMI, and NRS scores, were tested for normality using the Shapiro-Wilk test before analysis. As the data were normally distributed, comparisons between the two groups were made using independent sample t-tests. Categorical variables (such as sex distribution) were compared using the chi-square (χ²) test [19].

All results were presented as mean ± standard deviation (SD) for continuous variables and *n* (%) for categorical variables. Corresponding test statistics (t-values or χ²-values) and *P*-values were reported in the respective tables. A two-tailed *P*-value < 0.05 was considered statistically significant for all analyses.

## Results

**Figure 1:**
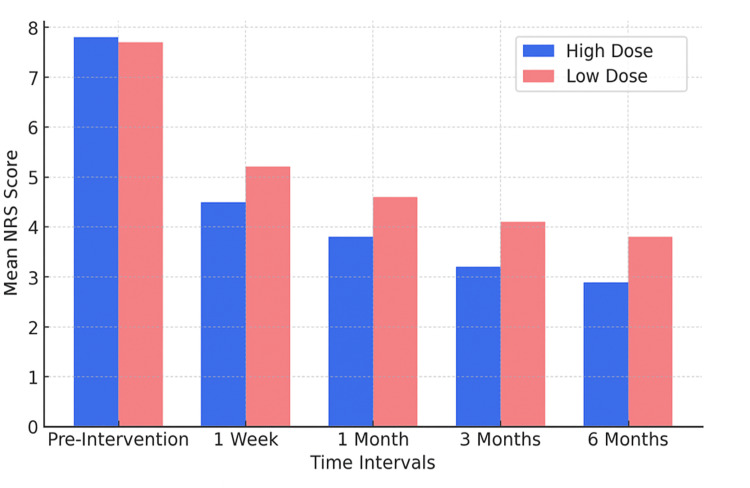
Numerical Rating Scale (NRS, 0-10) in both groups. It illustrates the trend in mean NRS pain scores over time in both the high-dose (HD) and low-dose (LD) groups. Both groups showed a steady decline in pain levels from baseline through the 6-month follow-up. However, the HD group consistently demonstrated lower mean NRS scores at all time points, indicating more rapid and sustained pain relief compared to the LD group.

**Figure 2:**
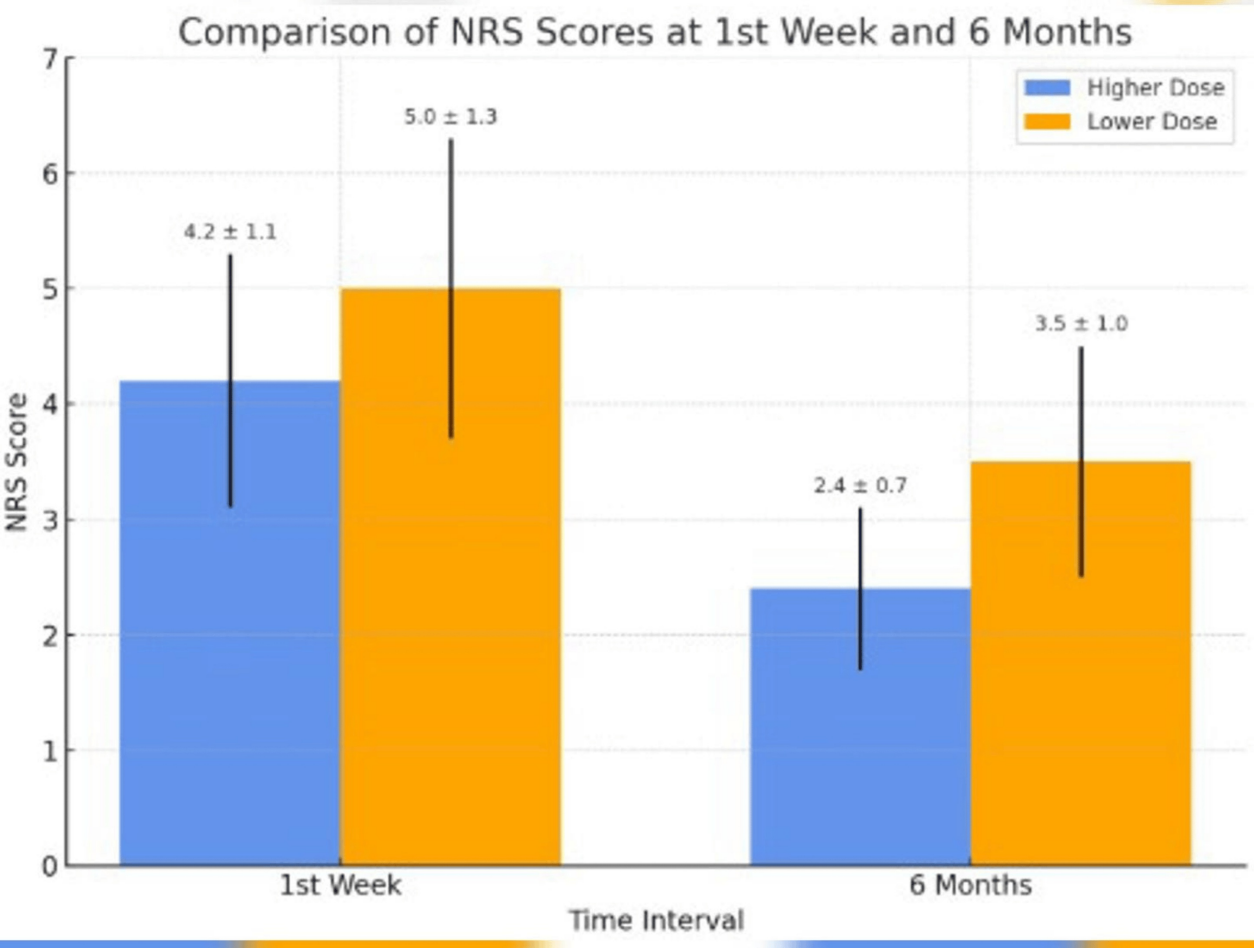
Comparison of NRS scores at the first week and six months. It provides a direct visual comparison of early and long-term outcomes between the groups. At the one-week mark, both groups experienced a significant reduction in pain, though the high-dose (HD) group had a larger absolute drop. By six months, the HD group maintained near-baseline pain control (NRS ~2.4), while the low-dose (LD) group exhibited a modest rebound (NRS ~3.5), suggesting a dose-dependent durability of effect. NRS, Numerical Rating Scale

Both groups were comparable with respect to age, sex, height, weight, and BMI (Table 1). Reduction in NRS scores (Table 2, Figures 1, 2) was better in Group HD compared to Group LD at one week, one month, three months, and six months post-procedure, all differences being highly significant (*P *< 0.001). Therefore, in our study, pain relief was there in both groups at six months, but it was better in Group HD compared to Group LD (*P *< 0.001). The 95% confidence interval (-1.68 to -0.52) for the mean difference, along with a Cohen’s d of 1.28, indicated a large effect size, suggesting that the difference in pain relief between the HD and LD groups at 6 months was not only statistically significant but also clinically meaningful. No adverse events or complications were observed in either group during the six-month follow-up period.

**Table 1:** Demographic parameters presented as mean ± standard deviation for continuous variables. *n* (%) for categorical variables. An independent t-test was used for continuous data, and the chi-square test was used for categorical data. Statistical significance was considered at *P* < 0.05.

Parameter	Group HD (mean ± SD) (*n *= 32)	Group LD (mean ± SD) (*n *= 32)	*t* /*χ*²-value	*P*-value
Age	54.5 ± 6.9 years	55.9 ± 5.6 years	*t* = 0.89	0.375
Height	164.4 ± 6.0 cm	165.3 ± 6.0 cm	*t* = 0.61	0.546
Weight	65.2 ± 7.0 kg	65.7 ± 6.8 kg	*t* = 0.29	0.772
BMI	24.1 ± 1.8	24.0 ± 1.6	*t* = 0.18	0.854
Sex (M:F)	11 (34.4%):21 (65.6%)	17 (53.1%):15 (46.9%)	*χ*² = 1.59	0.208

**Table 2:** Pain scores (NRS) comparison. Data were represented as mean ± standard deviation. An independent t-test was used for comparing groups, and statistical significance was considered at *P* < 0.05. NRS, Numerical Rating Scale

Time interval	Mean NRS score (higher dose) (*n *= 32)	Mean NRS score (lower dose) (*n* = 32)	*t*-value	*P*-value
Pre-intervention	8.1 ± 1.2	7.9 ± 1.3	*t* = 0.71	0.482
1 week post	4.2 ± 1.1	5.0 ± 1.3	*t* = 2.32	0.023
1 month post	3.5 ± 0.9	4.3 ± 1.2	*t* = 2.44	0.018
3 months post	2.8 ± 0.8	3.9 ± 1.1	*t* = 3.01	0.004
6 months post	2.4 ± 0.7	3.5 ± 1.0	*t* = 3.57	0.001

## Discussion

This study adds to the growing body of evidence supporting the use of ultrasound-guided corticosteroid injections in the management of subacromial bursitis. Our findings demonstrate that targeted delivery of corticosteroids significantly reduces pain intensity across all follow-up intervals, with more pronounced and sustained relief observed in the higher-dose group. This dose-response effect is consistent with the known pharmacological properties of corticosteroids, where higher intra-bursal concentrations produce more potent and lasting anti-inflammatory effects. The dose-dependent trend observed in this study supports the hypothesis that corticosteroids contribute meaningfully to symptom relief. These findings should be interpreted cautiously and serve as a basis for further randomized controlled trials with placebo arms to validate and refine the use of corticosteroids in subacromial bursitis.

Ultrasound guidance plays a pivotal role in improving the efficacy and safety of corticosteroid injections. Traditional blind techniques rely heavily on palpation and anatomical landmarks, which can lead to inaccurate delivery and suboptimal outcomes. In contrast, real-time ultrasound imaging ensures accurate needle placement and direct drug deposition into the subacromial bursa, thereby minimizing exposure to adjacent structures such as the rotator cuff tendons or joint capsule. This precision reduces the risk of iatrogenic injury, such as supraspinatus tendon damage or intratendinous injection, and enhances clinical results, as also reported by Akbari et al. [12]

The superior outcomes observed with the higher dose of triamcinolone (20 mg) in our study corroborate the findings of Sumanont et al. [18], who conducted a meta-analysis on corticosteroid volumes in subacromial bursitis. Their results suggested that higher steroid doses, particularly when combined with a local anesthetic, provided both immediate and sustained symptom relief. In our study, patients receiving the higher dose consistently reported lower pain scores at all intervals, including the six-month mark, suggesting that a larger steroid load contributes to longer suppression of the inflammatory cascade and delayed recurrence.

Subacromial bursitis often develops due to chronic microtrauma, impingement, or overuse, resulting in cytokine-mediated inflammation of the bursal tissue. Corticosteroids exert their effect by inhibiting phospholipase A2 and downregulating the synthesis of prostaglandins and leukotrienes, thereby attenuating both pain and inflammation at the cellular level. The presence of a local anesthetic such as lignocaine may further contribute to immediate pain relief, allowing patients to resume movement and prevent stiffness that may otherwise perpetuate the inflammatory cycle [20].

Our findings are also in agreement with the systematic review by van der Sande et al. [11], which concluded that corticosteroid injections are effective for short- to mid-term relief in subacromial impingement syndromes, including bursitis. While the benefits are clear, the study also emphasized the need for precise delivery techniques, something that ultrasound-guided approaches have helped to address. The improved outcomes in our study reinforce the importance of combining accurate technique with optimal dosing to maximize clinical benefit.

Despite the promising results, certain limitations must be acknowledged. First, the retrospective nature of the study introduces the potential for selection bias. While efforts were made to standardize inclusion and exclusion criteria, uncontrolled variables such as varying activity levels, occupation, and concurrent physiotherapy may have influenced outcomes. Additionally, the lack of randomization and blinding reduces the internal validity of the findings.

Another limitation is the relatively short follow-up period of six months. Although significant improvements were observed during this timeframe, it remains unclear whether the benefits of a single corticosteroid injection, especially at higher doses, are sustained beyond this period or whether repeat injections may be required. Long-term follow-up studies are needed to determine the durability of pain relief and to assess potential adverse effects such as tendon weakening, cartilage damage, or steroid-related complications with repeated dosing.

From a clinical perspective, our results offer several important implications. Firstly, ultrasound-guided corticosteroid injections appear to be a safe, effective, and minimally invasive treatment option for patients with subacromial bursitis who have failed conservative management. No serious adverse events or complications were observed in either group over the 6-month follow-up. Minor adverse events were infrequent and self-limiting:

Group HD (*n* = 32): 2 patients (6.3%) experienced mild post-injection soreness; 1 patient (3.1%) reported transient flushing.

Group LD (*n* = 32): 1 patient (3.1%) had localized soreness.

No infections, skin atrophy, tendon rupture, or systemic effects were reported in either group.

Second, the observed dose-dependent benefits suggest that steroid dosing should be individualized based on disease severity, chronicity, and patient comorbidities. While higher doses may offer longer-lasting relief, they must be weighed against the theoretical risks associated with corticosteroid exposure, particularly in physically active individuals or those with predisposing conditions like diabetes or osteoporosis.

Furthermore, the outpatient feasibility and cost-effectiveness of ultrasound-guided injections make them an attractive alternative to surgical interventions. Incorporating such ultrasound-guided procedures into standard interventional pain management practice could improve patient outcomes and reduce healthcare burden.

## Conclusions

This retrospective study underscores the clinical value of ultrasound-guided corticosteroid injections for managing subacromial bursitis, particularly in patients unresponsive to conservative therapy. Both LD and HD of triamcinolone offered significant pain relief; our findings support the effectiveness of corticosteroid injections for subacromial bursitis, with evidence favoring a higher dose for prolonged therapeutic effect over six months. The use of ultrasound not only enhanced the precision of drug delivery but also contributed to better patient outcomes by minimizing procedural complications and maximizing therapeutic efficacy.

The dose-dependent nature of corticosteroid response observed in this study supports the need for individualized treatment plans. Patients with more severe or chronic symptoms may benefit from higher doses, provided that potential risks are carefully evaluated. Importantly, no major adverse effects were noted during follow-up, affirming the safety profile of these interventions when performed under ultrasound guidance.

Despite inherent limitations such as its retrospective design and limited duration of follow-up, this study contributes meaningful insights to the existing literature. It highlights the importance of adopting standardized, evidence-based protocols for corticosteroid use in subacromial bursitis, especially in regions where such data are scarce. Future prospective, randomized trials with longer follow-up periods are necessary to further establish optimal dosing regimens and assess the long-term safety and effectiveness of repeated injections.

In summary, ultrasound-guided corticosteroid injection is a practical, effective, and safe intervention for subacromial bursitis, with higher doses offering more prolonged relief. This approach holds promise for improving patient quality of life while reducing dependence on more invasive or costly treatments.
